# DNA Barcoding Unveils Novel Discoveries in Authenticating High-Value Snow Lotus Seed Food Products

**DOI:** 10.3390/foods13162580

**Published:** 2024-08-18

**Authors:** Gang Zhao, Lingyu Li, Xing Shen, Ruimin Zhong, Qingping Zhong, Hongtao Lei

**Affiliations:** 1 Guangdong Provincial Key Laboratory of Food Quality and Safety, South China Agricultural University, Guangzhou 510642, China; gangzhao@scau.edu.cn (G.Z.); shenxing325@163.com (X.S.); zhongqp@scau.edu.cn (Q.Z.); 2 Guangdong Provincial Key Laboratory of Utilization and Conservation of Food and Medicinal Resources in Northern Region, Shaoguan University, Shaoguan 512005, China; zhongrm9898@163.com; 3 College of Plant Protection, South China Agricultural University, Guangzhou 510642, China; lilingyu7788@163.com

**Keywords:** *Gleditsia sinensis*, DNA barcoding, species identification, food fraud, Snow Lotus Seed

## Abstract

Snow Lotus Seed (SLS), esteemed for its nutritional and market value, faces challenges of authentication due to the absence of appropriate testing standards and methods. This results in frequent adulteration of SLS sourced from *Gleditsia sinensis* (*G. sinensis*) with other plant seeds endosperm. Traditional chloroplast DNA barcoding methods are inadequate for species identification due to the absence of chloroplasts in *G. sinensis* seeds endosperm. In this study, the homology of 11 ITS genes among 6 common *Gleditsia* species was analyzed. Universal primers suitable for these species were designed and screened. A DNA barcoding method for distinguishing SLS species was developed using Sanger sequencing technology, leveraging existing GenBank and Barcode of Life Data System (BOLD) databases. Optimized sample pretreatment facilitated effective DNA extraction from phytopolysaccharide-rich SLS. Through testing of commercial SLS products, the species origin has been successfully identified. Additionally, a novel instance of food fraud was uncovered, where the *Caesalpinia spinosa* endosperm was used to counterfeit SLS for the first time. The study established that the developed DNA barcoding method is effective for authenticating SLS species. It is of great significance for combating food fraud related to SLS, ensuring food safety, and promoting the healthy development of the SLS industry.

## 1. Introduction

Snow Lotus Seed (SLS), known as “Zao Jiao Mi” in Chinese, is a product made from the dried mature fruits of the artificially cultivated *Gleditsia sinensis*, through the processes of pod removal, seed extraction, soaking, steaming, endosperm extraction, and drying [1]. After soaking in water, SLS becomes translucent and resembles the Tian Shan snow lotus, which is how it earned its name. It is primarily used in the preparation of sweet soups and desserts and is mainly produced in China’s Guizhou and Yunnan provinces. The main component of SLS is oligosaccharides, and it is rich in plant dietary fiber and various minerals. With high energy and low fat, it is considered a healthy food choice and is well loved by many consumers [2]. Zhijin County in Guizhou Province is recognized as China’s largest processing center for SLS, with an annual processing and sales volume exceeding 1000 tons, capturing over 90% of the market share. Its products are widely acclaimed and distributed globally. SLS has also become an important characteristic economic agricultural product for local farmers in Zhijin County to abolish poverty and become rich [3,4]. However, due to the limited annual production of SLS, its production and processing currently depend largely on manual labor, leading to low processing efficiency. This is a major reason for its relatively high market price. As a result, some unethical traders substitute the endosperm of other plant seeds for SLS processing and sales, seeking substantial profits from this practice. The shape and color of these plant seed endosperms closely resemble those of *Gleditsia sinensis*. After being processed into finished SLS products, consumers find it even more difficult to accurately identify the species’ origin based solely on appearance. On one hand, this type of food fraud seriously undermines consumer interests and fosters the detrimental “Bad money drives out good money” phenomenon, hindering the healthy and sustainable development of the SLS industry [5]. On the other hand, commercially available SLS products derived from unidentified plant seeds may pose certain food safety risks. Currently, there have been no reported methods for authenticating the species of SLS products, making law enforcement against SLS food fraud increasingly challenging. Therefore, there is an urgent need to develop accurate methods for verifying its species authenticity.

In recent years, food adulteration detection technology has emerged as a prominent research focus within the global food industry. DNA-based molecular biology techniques have gained widespread recognition as highly accurate methods for species identification. Technologies such as PCR and its derivatives are extensively employed to verify the authenticity of plant-derived species like coffee and fruit juices [6,7]. Researchers have also utilized methods such as SRAP, SSR, transcriptome analysis, and genome sequencing for identifying plant varieties and distinguishing between male and female plants [8,9,10,11]. However, these approaches often suffer from issues such as instability, complex analyses, cumbersome procedures, or high costs. Real-time PCR, considered the gold standard for biological species identification, excels in targeted identification of specific species but faces challenges in identifying unknown species [12]. With the advancement and widespread adoption of Sanger sequencing technology, DNA barcoding has emerged as a powerful tool for taxonomic studies, especially for identifying unknown species [13]. Chloroplast DNA (cpDNA) is particularly suitable for species differentiation due to its matrilineal inheritance in most plants and slow evolutionary rate [14,15]. Several cpDNA genes such as *rbcL*, *matK*, *psbA*-*trnH*, and *ycf1b* have been identified as effective targets for DNA barcoding to accurately distinguish plant species [16,17,18,19]. Among these, the *rbcL* and *matK* genes are established targets for standard plant species barcodes recognized by the Barcode of Life Data System (BOLD) [20]. However, as the endosperm of SLS contains abundant plant polysaccharides, its DNA content is naturally low and lacks chloroplast DNA. Processing steps such as steaming and drying further exacerbate DNA fragmentation and loss. Moreover, many traditional universal DNA barcodes for plants amplify long target segments (600~1200 bp) [21,22], with most targeting chloroplast genes, thus they are not suitable for species identification of SLS raw materials and commercial products.

**Figure 1:**
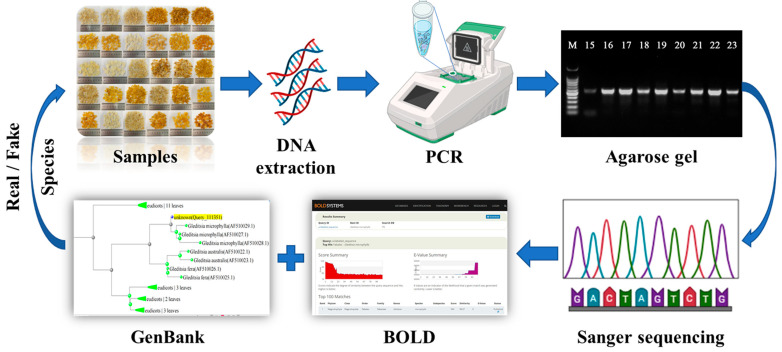
DNA barcoding assay for the authentication of commercially available SLS products.

Hence, in order to address the challenge of lacking available methods for species identification of raw materials and commercial products of SLS, this study constructed a DNA barcoding method for identifying the species origin of SLS by analyzing the sequence information from the ITS region of nuclear genes in various common *Gleditsia* species (Figure 1). Through testing, this method has good amplification performance and differentiation ability for common *Gleditsia* species and can be used for species identification of raw materials and commercially available products of SLS. This study offers technical support for verifying the authenticity of SLS species, which is crucial for combating food fraud, safeguarding consumer interests, and promoting the green, healthy, and sustainable development of SLS and its associated industries.

## 2. Materials and Methods

### 2.1. Samples

**Figure 2:**
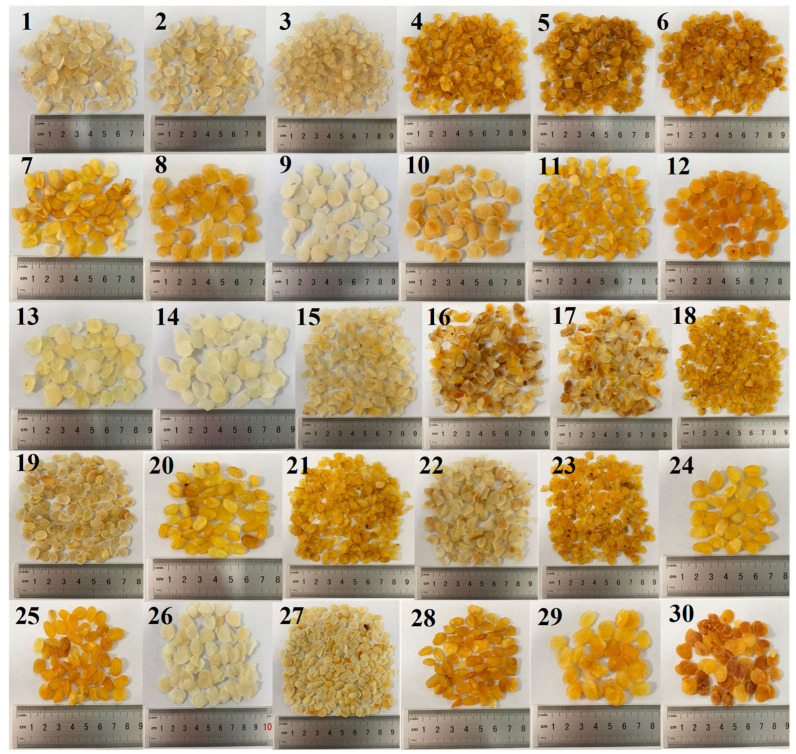
Thirty commercially available SLS products collected for this study.

The seeds of *Gleditsia sinensis*, *Gleditsia japonica*, and *Gleditsia microphylla* were obtained from Guangdong Provincial Key Laboratory of Food Quality and Safety. *Gleditsia delavayi* seeds were procured from the *Gleditsia delavayi* planting base (Meihui *Gleditsia delavayi* planting Base, Lianghe, China). *Gleditsia fera* seeds were collected from South China Agricultural University. After washing all seeds twice with sterilized water, they were soaked overnight in water at room temperature. Subsequently, the endosperm extracted by dissecting the seed coat with a surgical blade was dried at 60 °C in a DHG 9420(A) electric forced air-drying oven (Bluepard instruments Ltd., Shanghai, China) for 4 h [23]. Finally, the endosperm samples were stored at −20 °C. The 30 samples of commercially available SLS were procured from Yunnan (Samples 1~5), Guizhou (Samples 6~10), Shaanxi (Samples 10~15), Henan (Samples 15~20), Hebei (Samples 20~25), and Shandong (Samples 25~30) provinces (Figure 2).

### 2.2. DNA Extraction

The DNA was extracted from SLS samples using the DP360 Plant DNA Extraction Kit (Tiangen Biochemical Technology Ltd., Beijing, China), and the instructions were improved for use. Briefly, SLS samples were pulverized by a WFB-D1 wall-breaker (Westinghouse Electric Ltd., Ningbo, China) and passed through an 80-mesh sieve to remove large particles that were not completely pulverized. A 20 mg powder sample was mixed with 800 μL of lysis buffer and 20 μL of RNase A (10 mg/mL) in an EP tube at 65 °C for 10 min. Subsequently, 200 μL neutralization buffer was added to the tube and mixed thoroughly and place it on ice for 10 min. The sample was placed on ice for 10 min, and then the procedure was followed as described in the product manual until the sample DNA was obtained. Each sample was repeated 3 times and the DNA obtained was placed at −20 °C for storage.

### 2.3. Primer and PCR Amplification

**Figure 3:**
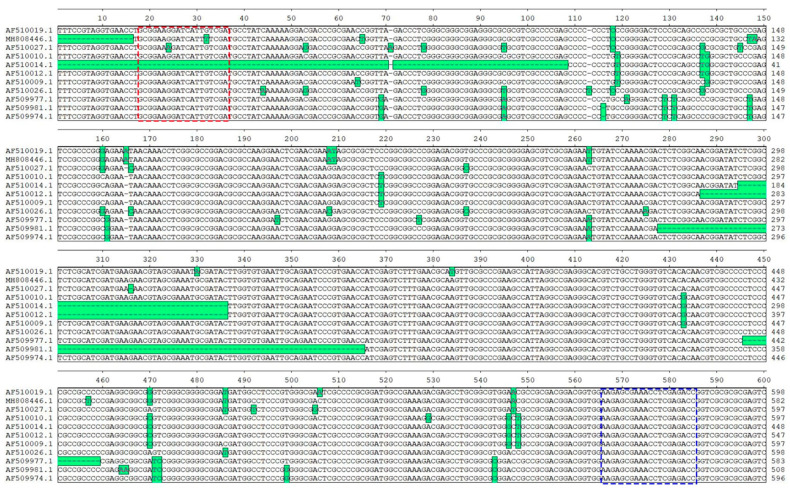
The homology analysis for 11 ITS genes from 6 *Gleditsia* species. The red and blue dashed boxes are the DNA barcoding forward and reverse primer design regions, respectively. Differential bases are labeled in green. The accession numbers of the ITS genes from top to bottom in the image belong to the following species: *Gleditsia sinensis*, *Gleditsia sinensis*, *Gleditsia microphylla*, *Gleditsia japonica*, *Gleditsia japonica*, *Gleditsia japonica*, *Gleditsia delavayi*, *Gleditsia fera*, *Gleditsia triacanthos*, *Gleditsia triacanthos*, *Gleditsia triacanthos*.

A total of 11 ITS genes from *Gleditsia sinensis* (MH808446.1, AF510019.1), *Gleditsia japonica* (AF510012.1, AF510014.1, AF510010.1), and *Gleditsia microphylla* (AF510027.1), *Gleditsia triacanthos* (AF509977.1, AF509981.1, AF509974.1), *Gleditsia delavayi* (AF510009.1) and *Gleditsia fera* (AF510026.1) six *Gleditsia* species were downloaded from GeneBank database. Sequence homology analysis was performed using the “Clustal W Method” in MegAlign software version 7.1.0 (DNASTAR, Inc., Madison, WI, USA), and the conserved regions were selected for the design of generic DNA barcoding primers for *Gleditsia* (Figure 3). The four most common *Gleditsia* seed endosperm DNAs, *Gleditsia sinensis*, *Gleditsia japonica*, *Gleditsia microphylla*, and *Gleditsia delavayi*, were used for generalization testing of SLS DNA barcoding primers. Common plant DNA barcoding universal primers for ITS gene were also used for comparative suitability testing of SLS [24,25,26]. The PCR reaction was performed in 50 μL containing 41 μL of 1.1× T6 Super PCR Mix (Tsingke Biotech Ltd., Beijing, China), 2 μL of 10 μM each forward and reverse primer, and 5 μL of DNA template (10 ng/μL). The thermal cycling parameters were shown as follows: pre-denaturation at 98 °C for 2 min, followed by 39 cycles of 98 °C for 10 s, 56 °C for 15 s, and 72 °C for 15 s with a final extension at 72 °C for 5 min. Sterile ultrapure water as a template was used as a negative control to ensure that the PCR reaction was not contaminated. All primers (Table 1) were synthesized by GENEWIZ (GENEWIZ Biotechnology Ltd., Suzhou, China).

### 2.4. Sanger Sequencing

The above PCR amplification products and 100 bp DNA ladder (Tsingke Biotech Ltd., Beijing, China) were electrophoresed on a 2% agarose gel to determine the size of the bands. After confirming the successful amplification, the amplified products, together with the corresponding amplification primers, were submitted to GENEWIZ Biotechnology Ltd. for Sanger sequencing using the standard procedure of ABI 3730 DNA sequencing platform. To ensure the accuracy of the sequencing results, the PCR products were sequenced in both directions in this study. Sequencing results were returned by DNASTAR. Lasergene.v7 software (DNASTAR, Inc., Madison, WI, USA) for sequence splicing and manual sequence correction when necessary.

### 2.5. Phylogenetic Analysis

The acquired sequences were first subjected to homology analysis using the BLAST tool [27]. The phylogenetic analysis was conducted using the following BLAST tool workflow: the corrected sequence was inputted and searched against the nucleotide collection (nr/nt) within standard databases. The “Organism” field was specified as “plants”, while other parameters were maintained at default settings. Sequence comparison was performed using the “Highly similar sequences (megablast)” algorithm in the Program Selection column. Subsequently, a Max Seq Difference of 0.75 was applied based on the BLAST results to construct a phylogenetic tree using the Neighbor Joining method [28].

This study also utilized the Barcode of Life Data System (BOLD) [29] to conduct secondary homology analysis on the acquired sample sequences, thereby reaffirming the origin of the analyzed specimens. Due to the limited gene targets, *rbcL* and *matK*, located exclusively in chloroplast DNA, the barcode database (Plant identification) within the BOLD System is insufficient for identifying SLS, which lacks chloroplast DNA. Consequently, corrected sequences from SLS samples were submitted to the “FUNGAL IDENTIFICATION [ITS]” database within the “IDENTIFICATION ENGINE” module for homology analysis.

## 3. Results and Discussion

### 3.1. DNA Barcoding Primers for SLS

**Figure 4:**
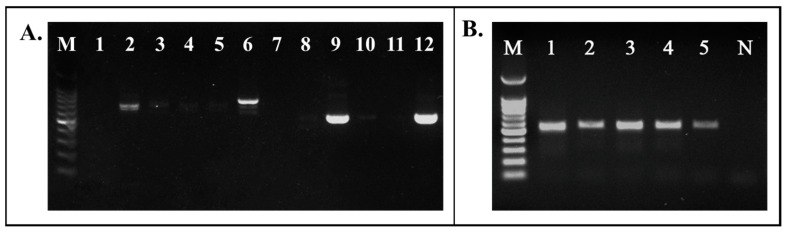
DNA barcoding primers were tested for amplification versatility against common *Gleditsia* species. (**A**) Amplification performance of primers ITS-F and ITS-R, and ITS2-F and ITS2-R on DNA of different species of SLS. Numbers 1–6: ITS-F and ITS-R were used for amplification. Numbers 7–12: ITS2-F and ITS2-R were used for amplification. M: 100 bp DNA Ladder. Numbers 1–6 and 7–12: No template control, *Gleditsia sinensis*, *Gleditsia microphylla*, *Gleditsia japonica*, *Gleditsia delavayi*, and *Gleditsia fera*. (**B**) DNA from different species of SLS were tested for amplification versatility ability of primers G-F and G-R. M: 100 bp DNA Ladder. Numbers 1–4: DNA templates were obtained from *Gleditsia sinensis*, *Gleditsia microphylla*, *Gleditsia japonica*, and *Gleditsia delavayi*, respectively. Number 5: *Gleditsia fera*. N: No template control.

The widely cited plant DNA barcoding universal primer pairs ITS-F/ITS-R and ITS2-F/ITS2-R were employed in this study to assess their amplification capability across five common varieties of SLS. Through multiple amplification tests, it was observed that both primer pairs exhibited limited universal amplification ability for SLS varieties sourced from the market (Figure 4A). Specifically, ITS-F/ITS-R showed better recognition and amplification performance for SLS DNA from *G. sinensis* and *G. fera*, but struggled with DNA from *G. microphylla*, *G. japonica*, and *G. delavayi*. Similarly, ITS2-F/ITS2-R effectively amplified SLS DNA from *G. microphylla* and *G. fera*, but encountered challenges in recognizing and amplifying DNA from *G. sinensis*, *G. japonica*, and *G. delavayi*. Upon analysis of this primer with *Gleditsia*, these two sets of reported universal primer pairs for plant DNA barcoding differed to some extent from the ITS gene sequences of *Gleditsia*, resulting in insufficient recognition and binding of different DNAs from *Gleditsia*, making it difficult to achieve universal amplification of SLS DNAs from common species sources. Thus, by reanalyzing the homology of 11 ITS genes of 6 *Gleditsia* species, this study designed and screened to obtain DNA barcoding universal amplification primers: G-F and G-R, suitable for common species of SLS in the market. Through testing, the G-F and G-R primers can better identify the DNA of five common *Gleditsia* species originating from SLS in the market, and all of them can obtain a single electrophoretic band amplicon of about 550 bp in size (Figure 4B), which can be used for Sanger sequencing and subsequent species analysis.

### 3.2. The Authenticity of Commercial SLS Products

**Figure 5:**
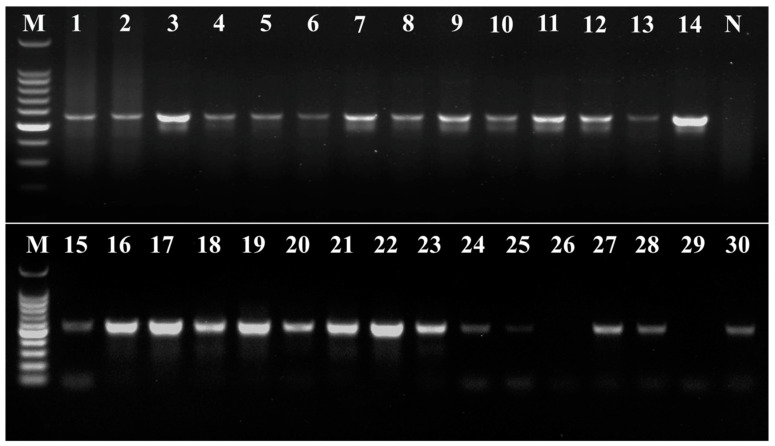
DNA barcoding universal primers for amplification assay of 30 commercial SLS products sold in the market. The amplicons of 30 SLS products using DNA barcoding primers G-F and G-R. Numbers 1–30: 30 commercial SLS products. M: 100 bp DNA Ladder. N: No template control.

After 30 commercial SLS products were sequenced by Sanger, the sequence results were verified by simultaneous comparison with both GenBank and BOLD databases, and the species with the highest homology and consistent results between the two databases were selected as the species to which the SLS products belonged. The results showed that 28 of the samples successfully obtained target amplicons and completed Sanger sequencing and subsequent analysis (Figure 5). Two samples (26 and 29) failed to yield PCR amplification despite multiple attempts at DNA extraction, primarily because successful DNA sample acquisition was never achieved for these samples. Additionally, alternative DNA extraction methods using Magnetic Particle Adsorption (CZ307, Biomed Biotech, Beijing, China) and CTAB precipitation [30] were also employed on samples 26 and 29, but these methods also yielded minimal results. After observing and sensory tasting of the two samples, it was found that both samples had noticeably larger shapes and sweeter tastes, indicating they were artificially manufactured SLS with added sucrose. Our analysis suggests that it is highly likely that the use of additives such as sucrose and edible gel during processing further prevented the already limited DNA in the samples from being released normally.

Of the 28 successfully sequenced SLS products, only 8 samples were confirmed to be of true *G. sinensis* origin after comparison of the 2 databases, amounting to less than one-third (26.6%) of the total samples (Table 2). Nearly two-thirds of the other number of products originated from *G. microphylla* (nine samples) and *G. japonica* (nine samples), which is a food fraud in the form of impersonation by a closely related species. Surprisingly, two samples (3 and 14) were found to not belong to any Gleditsia species based on the results of this sequencing analysis. The target sequences of these samples showed 100% homology with *Caesalpinia spinosa* in the BOLD database and with *Tara spinosa* in GenBank. Since *Tara spinosa* is a synonym of *Caesalpinia spinosa*, which belongs to the genus *Caesalpinia*, this represents food fraud involving species from a different genus. In conclusion, more than two-thirds of the 30 SLS products analyzed in this study were processed from seed endosperm of plants belonging to the same genus or different genera with *G. sinensis*. This widespread practice indicates prevalent food fraud involving SLS, highlighting the critical need to address the authenticity of SLS products.

### 3.3. New Discovery for Authenticity Identification of SLS

**Figure 6:**
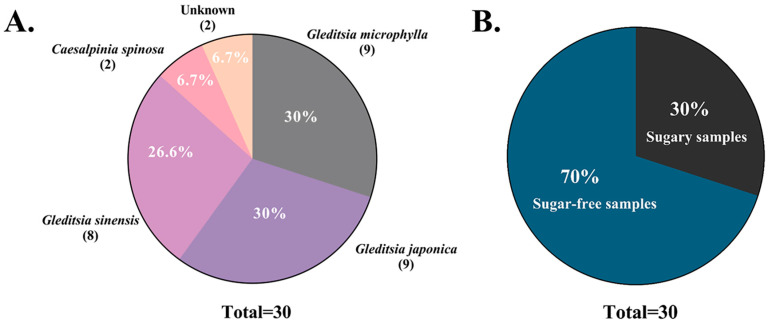
The authenticity of 30 commercial SLS products sold in the market. (**A**) Statistical results of species identification and classification of 30 commercially available SLS products. Two samples (26 and 29) failed to identify the species, accounting for 6.7% of the total (Unknown). The remaining samples identified the species as *Gleditsia sinensis*, *Gleditsia microphylla*, *Gleditsia japonica,* and *Caesalpinia spinosa*, accounting for 26.6%, 30%, 30%, and 6.7% of the total tested samples, respectively. (**B**) Based on sensory tasting, 30% (9 out of 30) of the SLS products were found to be adulterated with added sugar for increased weight, while the remaining 70% (21 out of 30) were products without added sugar.

After identifying the species origin of 30 commercial SLS products by the DNA barcoding method established in this study, only 26.6% of the samples were authentic SLS of *G. sinensis* origin, and the remaining samples were typically economically motivated adulteration (EMA) (Figure 6). Based on our field research at the SLS processing and distribution center in Guizhou, we believe there are currently three main instances of food fraud in the SLS products sold in the market: adulteration with closely related species belonging to the *Gleditsia* genus, misrepresentation of species such as *Caesalpinia spinosa* that do not belong to the *Gleditsia* genus, and the production of artificially enhanced SLS using sucrose and other additives for weight gain purposes.

#### 3.3.1. Adulteration of Closely Related Species of the Same Genus

Through our analysis of commercially available SLS products, we discovered that the endosperms from *Gleditsia microphylla* and *Gleditsia japonica* were frequently used to adulterate SLS, deceiving consumers into believing it was genuine *Gleditsia sinensis*. These adulterated samples, using closely related species to impersonate *G. sinensis*, accounted for 60% (*Gleditsia microphylla* 30% and *Gleditsia japonica* 30%) of the samples tested and represent a significant form of food fraud in the SLS market today (Figure 6A). *G. microphylla*, *G. japonica*, and *G. sinensis* are closely related species within the same genus, sharing similar genetic relationships and exhibiting very similar shapes and colors in their endosperms. This similarity makes it challenging to distinguish the origin of the species, whether in raw material form or as processed SLS products. Additionally, because the nursery costs for *G. microphylla* are significantly lower, only one-tenth of those for *G. sinensis* [31], and *G. japonica* seeds are smaller with less endosperm fullness, the prices of both *G. microphylla* and *G. japonica* seeds are lower compared to *G. sinensis* seeds. This has led to some growers and unscrupulous merchants selling seeds from *G. microphylla* and *G. japonica* disguised as *G. sinensis* seeds for the production of high-value SLS to make a profit. While the endosperms of *G. microphylla* and *G. japonica* also contain a considerable amount of plant polysaccharides and exhibit a translucent, gelatinous state when soaked in water, whether these endosperms can be safely consumed as food has not been definitively reported. Therefore, the practice of using closely related species from the genus *Gleditsia* to masquerade as *G. sinensis*-origin SLS not only infringes upon consumers’ rights to accurate information but also poses potential food safety risks. Overall, this behavior highlights the need for stricter oversight and authentication measures in the SLS industry to ensure transparency and consumer safety.

#### 3.3.2. Caesalpinia Spinosa: A Newly Identified Non-Gleditsia Species Involved in Adulterating SLS

In this study, 2 (sample 3 and 14) of the 30 commercial SLS products were identified as belonging to the *Caesalpinia spinosa* (*Tara spinosa*), accounting for 6.7% of the total samples (Figure 6A). It is distantly related to *G. sinensis* and belongs to a different genus in the *Fabaceae*. However, the *Caesalpinia spinosa* has pods with a similar appearance to the *G. sinensis*, and the seed endosperm is also rich in phytopolysaccharides, which are not directly distinguishable from SLS after processing. *Caesalpinia spinosa* is native to Peru and Ecuador in South America, and its seeds are the raw material for the production of the edible Tara gum [32,33]. Because of the large area under cultivation and the high yield and cheap price of the seeds of a single plant, there are unscrupulous merchants who use the endosperm of *Caesalpinia spinosa* seeds to process and pretend to be high-value SLS to obtain huge profits. Although the Tara gum produced from its seeds can be used as a food additive, it does not mean that the entire seed endosperm can be used directly as an edible part. Therefore, utilizing *Caesalpinia spinosa* seed endosperms in the production of SLS products still carries some undisclosed risks, greatly infringing upon consumers’ right to knowledge and economic interests.

#### 3.3.3. Artificially Manufactured Weighted SLS with Added Sucrose

Combined with the previous field research and the analysis of this commercially available samples of SLS, we also found food fraud in samples where sugar and other additives were mixed with low-grade or small SLS to increase the weight of the product for profit. In the blind SLS samples test, nine samples (sample 8, 9, 10, 12, 13, 14, 26, 29, 30) intentionally had sugar added to increase product weight, accounting for as much as 30% of the total samples (Figure 6B). These sugared samples were all larger than the common SLS form, and the surface was not smooth, sticky to the touch, and sweet to the taste when licked directly. Two of these samples, moreover, were so likely to be due to, for example, excessive sugar adulteration, that the sample DNA could not be successfully extracted for subsequent analysis. In addition, the authors have made several field research trips to Maochang Town in Zhijin County, the largest SLS processing and distribution center in China. Through exchanges with local SLS manufacturing workers and wholesalers, it was indirectly confirmed that the use of white granulated sugar to adulterate SLS has become one of the most important means for some unscrupulous traders to obtain huge profits. As the price of sugar (~0.8 USD/kg) is much lower than the market price of SLS (60 USD/kg), the difference between the two is more than 60 times, and the use of sugar processing to obtain sugary SLS can be made to make SLS weigh 2–5 times than its original weight, leading to profit. This artificially weighted SLS, although it uses edible white sugar, is not only a consumer fraud, but a criminal offense against consumers expecting to stay in shape or for patient populations on low-sugar diets.

## 4. Conclusions

In this study, we developed a DNA barcoding method for the identification of species adulteration in raw materials for the production of high-value SLS and commercial products by designing ITS universal amplification primers. The method has good target identification and amplification performance for common *G. sinensis*, *G. microphylla*, *G. japonica*, *G. delavayi*, and *G. fera* species. By analyzing both GenBank and BOLD databases simultaneously, SLS from *G. microphylla* and *G. japonica* sources were accurately distinguished as *G. sinensis* sources. Moreover, the fraudulent use of the endosperm of the seed of a non-*Gleditsia* plant as SLS was also accurately identified. Through testing of commercial SLS products, this study found that more than 70% of the products were adulterated to varying degrees. Building on authors’ previous field investigations, this study systematically reports, for the first time, three major kinds of food adulteration targeting high-value SLS products. The first new finding of food fraud in which the seed endosperm of *Caesalpinia spinosa*, a plant species of a different genera, was used for processing and was passed off as SLS. This study fills the gap in the authenticity identification method of high-value SLS species. It provides technical support to confirm the authenticity of raw material sources for SLS-processing enterprises, as well as for food regulatory-related agencies to combat SLS species fraud. It is also hoped that the establishment of the methodology in this study will draw the attention of the relevant food legislature to the establishment of relevant analytical and certification standards for high-value SLS so that healthier and safer SLS-related foods can be developed. This will promote the healthy and sustainable development of the SLS industry, and at the same time, it will also enable the consumers to obtain more protection for their health and economic interests.

## Figures and Tables

**Table 1:** Information of the oligonucleotides used in this work.

Primer	Oligonucleotide (5′-3′)	Amplicon (bp)	Reference
G-F	GCGGAAGGATCATTGTCGA	550	This study.
G-R	GGTCTCGAGGTTTCGCTCTT		
ITS-F	GGAAGTAAAAGTCGTAACAAGC	731	[25]
ITS-R	TCCTCCGCTTATTGATATGC		
ITS2-F	ATGCGATACTTGGTGTGAAT	450~550	[24]
ITS2-R	GACGCTTCTCCAGACTACAAT		

**Table 2:** DNA barcode sequencing results of 30 SLS samples were compared with information from GenBank and BOLD databases.

Sample No.	GenBank				BOLD			Species Judgment ^a^
	Species	Accession	Total Score	Similarity (%)	Species	Score	Similarity (%)	
1	*Gleditsia sinensis*	AF510020.1	1020	100	*Gleditsia sinensis*	548	99.64	R
2	*Gleditsia sinensis*	AF510020.1	1011	99.82	*Gleditsia sinensis*	543	99.63	R
3	*Tara spinosa*	OQ411711.1 OQ411709.1	872	100	*Caesalpinia spinosa*	472	100	F
4	*Gleditsia microphylla*	AF510027.1	667	100	*Gleditsia microphylla*	359	99.72	F
5	*Gleditsia microphylla*	AF510027.1	985	99.63	*Gleditsia microphylla*	533	98.9	F
6	*Gleditsia microphylla*	AF510027.1	662	100	*Gleditsia microphylla*	356	99.72	F
7	*Gleditsia sinensis*	AF510020.1	937	99.61	*Gleditsia sinensis*	504	99.41	R
8	*Gleditsia japonica*	MH710914.1	1002	99.82	*Gleditsia japonica*	539	100	F
9	*Gleditsia japonica*	MH710914.1	996	99.82	*Gleditsia japonica*	540	99.82	F
10	*Gleditsia microphylla*	AF510027.1	667	100	*Gleditsia microphylla*	359	99.72	F
11	*Gleditsia japonica*	MH710914.1	675	100	*Gleditsia japonica*	365	100	F
12	*Gleditsia japonica*	MH710914.1	680	100	*Gleditsia japonica*	368	100	F
13	*Gleditsia microphylla*	AF510027.1	665	99.73	*Gleditsia microphylla*	357	99.72	F
14	*Tara spinosa*	OQ411710.1	713	100	*Caesalpinia spinosa*	475	99.38	F
15	*Gleditsia japonica*	MH710914.1	1000	100	*Gleditsia japonica*	541	100	F
16	*Gleditsia japonica*	MH710914.1	1013	100	*Gleditsia japonica*	548	100	F
17	*Gleditsia japonica*	MH710914.1	1005	100	*Gleditsia japonica*	544	100	F
18	*Gleditsia microphylla*	AF510027.1	1005	99.64	*Gleditsia microphylla*	543	99.27	F
19	*Gleditsia sinensis*	AF510020.1	1024	100	*Gleditsia sinensis*	550	99.64	R
20	*Gleditsia sinensis*	AF510020.1	1013	100	*Gleditsia sinensis*	544	99.64	R
21	*Gleditsia microphylla*	AF510027.1	1000	99.82	*Gleditsia microphylla*	539	99.45	F
22	*Gleditsia japonica*	MH710914.1	1005	99.64	*Gleditsia japonica*	545	99.82	F
23	*Gleditsia microphylla*	AF510027.1	1005	99.82	*Gleditsia microphylla*	542	99.45	F
24	*Gleditsia sinensi*	AF510020.1	833	99.56	*Gleditsia sinensis*	449	99.12	R
25	*Gleditsia sinensis*	AF510020.1	992	100	*Gleditsia sinensis*	533	99.63	R
26	/	/	/	/	/	/	/	N
27	*Gleditsia japonica*	MH710914.1	1014	100	*Gleditsia japonica*	549	100	F
28	*Gleditsia sinensi* *s*	AF510020.1	1003	100	*Gleditsia sinensis*	539	99.63	R
29	/	/	/	/	/	/	/	N
30	*Gleditsia microphylla*	AF510027.1	990	99.63	*Gleditsia microphylla*	535	99.26	F

**^a^** R: Real, the sample is sourced from *Gleditsia sinensis.* F: Fake, the sample is not sourced from *Gleditsia sinensis.* N: Sequencing was incomplete.

## Data Availability

The original contributions presented in the study are included in the article and supplementary material, further inquiries can be directed to the corresponding author.

## Supplementary Materials

The following supporting information can be downloaded at: https://www.mdpi.com/article/10.3390/foods13162580/s1, The results (5′-3′) of Sanger sequencing for 30 Snow Lotus Seed products amplified using G-F/G-R primers.
