# Supplementary material for: DNA Barcoding Unveils Novel Discoveries in Authenticating High-Value Snow Lotus Seed Food Products
Source: Foods. 2024 Aug 18;13(16):2580. doi: 10.3390/foods13162580 (PMC11353642; doi:10.3390/foods13162580)
Supplement: Supplementary file 1 [file foods-13-02580-s001.zip › foods-3114522-supplementary.pdf]

DNA barcoding unveils novel discoveries in authenticating high-value Snow Lotus Seed food products

Gang Zhao [a, b], Lingyu Li [c], Xing Shen [a], Ruimin Zhong [b], Qingping Zhong [a], Hongtao Lei [a, *]

[a] *Guangdong Provincial Key Laboratory of Food Quality and Safety, South China Agricultural University, Guangzhou 510642, China*

[b] *Guangdong Provincial Key Laboratory of Utilization and Conservation of Food and Medicinal Resources in Northern Region, Shaoguan University, Shaoguan 512005, China*

[c] *College of Plant Protection, South China Agricultural University, Guangzhou 510642, China*

*Corresponding author

Tel.: +8620 8528 3925; Fax: +8620 8528 0270

E-mail: hongtao@scau.edu.cn

Address: 483 Wushan Road, Tianhe District, Guangzhou 510642, China.

The results (5’-3’) of Sanger sequencing for 30 Snow Lotus Seed products amplified using G-F/G-R primers.

Sample 1:

GATCATTGTCGATGCCTATCAAAAAGGACGACCCGCGAACCGGTTAGACCC
TCGGGCGGGCGGAGGGCGCGCGTCGCCCCGAGCCCCCCCTCCCGGGGACT
CCCGCAGCCCCGCGCTGCCCGAGTCCGCCCGGGAGAAATAACAAACCTCG
GCGCCGGACGCGCCAAGGAACTCGAACGAAATAGCGCGCTCCCGGCGGCC
CGGAGACGGTGCCCGCGCGGGGAGCGTCGCGAGAATTGTATCCAAAACGA
CTCTCGGCAACGGATATCTCGGCTCTCGCATCGATGAAGAACGTAGCGAAA
TGCGATACTTGGTGTGAATTGCAGAATCCCGTGAACCATCGAGTCTTTGAA
CGCAAGTTGCGCCCGAAGCCATTAGGCCGAGGGCACGTCTGCCTGGGTGT
CACACAACGTCGCCCCCTCCCCGCCGCCCCCGAGGCGGCGGGTCGGGCGG
GGCGGATGATGGCCTCCCGTGGGCGAATCGCCCCGCGGATGGCCGAAAGA
CGAGCCTGCGGCGTGGAACGCCGCGACGGACGGTGGAAGAGCGAAACCT

Sample 2:

AGGTTTCGCTCTTCCACCGTCCGTCGCGGCGTTCCACGCCGCAGGCTCGTC
TTTCGGCCATCCGCGGGGCGATTCGCCCACGGGAGGCCATCATCCGCCCCG
CCCGACCCGCCGCCTCGGGGGCGGCGGGGAGGGGGCGACGTTGTGTGAC
ACCCAGGCAGACGTGCCCTCGGCCTAATGGCTTCGGGCGCAACTTGCGTTC
AAAGACTCGATGGTTCACGGGATTCTGCAATTCACACCAAGTATCGCATTT
CGCTACGTTCTTCATCGATGCGAGAGCCGAGATATCCGTTGCCGAGAGTCG
TTTTGGATACAATTCTCGCGACGCTCCCCGCGCGGGCACCGTCTCCGGGCC
GCCGGGAGCGCGCTATTTCGTTCGAGTTCCTTGGCGCGTCCGGCGCCGAGG
TTTGTTATTTCTCCCGGGCGGACTCGGGCAGCGCGGGGCTGCGGGAGTCCC
CGGGAGGGGGGGCTCGGGGCGACGCGCGCCCTCCGCCCGCCCGAGGGTC
TAACCGGTTCGCGGGTCGTCCTTTTTGATAGGCATCGACAAATGAAA

Sample 3:

GTGTGAATTGCAGAATCCCGTGAACCATCGAGTCTTTGaACGCAAGTTGCG
CCCGAAGCCATTAGGCCGAGGGCACGTCTGCCTGGGCGTCACACATCGTTG
CCCCCCCAACCACCGTGCCTCGCAAGAGGACGTGACGGGATGGGCGGATC
ATGGCCTCCCGCGGGCCTCGTCTCGCGGCTGGCCGAAATAAGAGTCCTCGG
TGGCGGTCGCCACGCTCCTCGGTGGACGAGTTTAACTCGATGCCGGTCGTG
CGCGCGCCGTCACCTGGGCCGGGCTCGGAGACCCTGTCGTGTCGCCCCGA
GCGACAACGAAACCAACGCGACCCCAGGTCAGGCGGGGCTACCCGCTGA
GTTTAAGCATATCAATAAGCGGAGGAAAAGAAACTAACAAGGATTCCCCTA
GTAACGGCGAGCGAACCGGGAAAAGCCCACCATGAAAATCGgTCGCCCCA
GGCGTCCGAATTGTAGTCTG

Sample 4:

TGGTGTGAATTGCAGAATCCCGTGAACcaTCGAGTCTTTGAACGCAAGTTG
CGCCCGAAGCCATTAGGCCGAGGGCACGTCTGCCTGGGTGTCACACAACG
TCGCCCCCTCCCCGCCGCCCCCGAGGCGGCGAGTCGGGCGGGGCGGATGA
TGGTCTCCCGTGGGCGGCTCGCCCCGCGGATGGCCGAAAGACGAGCCTGC

GGCGTGGATCGCCGCGACGGACGGTGGAAGAGCGAAACCTCGAGACCGG
TCGCGCGCGAGTCCTCCCTTGGGGCAGGCTGCCAGACCCTTTAGCGTCGCC
GCGTACGGCGGGCGCTTACGACGCGACCCCAGGTCAGGCGGGGCTACCCG
CTGAGTTTAAGCATATCAATAAGCGGAGGAAAAGAAACTAACCAGGATTCC
CTTAGTAACGGCGAGCGAACCGGGAAAAGCCCACCTtGAGAATCGGTCGC
CCCCGGCGTCCGAATTGTAGTCT

Sample 5:

TTTTTGATGCCTATCAAAAAGGAGGACCCGCGAACCGGTTAAGACCCTGGG
GCGGGCGGAGGGCGTGCGTCGCCCCGAGCCCCCCCTCCCGGGGACTCCCG
CAGCCGCGCGCTGCCCGAGTCCGCCCGGGAGAACAACAAACCTCGGCGCC
GGACGCGCCAAGGAACTCGAACGAAGGAGCGCGCTCCCGGCGGCCCGGA
GACGGCGCCCGCGCGGGGAGCGTCGCGAGAACTGTATCCAAAACGACTCT
CGGCAACGGATATCTCGGCTCTCGCATCGATGAAGAACGTAGCGAAATGCG
ATACTTGGTGTGAATTGCAGAATCCCGTGAACCATCGAGTCTTTGAACGCA
AGTTGCGCCCGAAGCCATTAGGCCGAGGGCACGTCTGCCTGGGTGTCACA
CAACGTCGCCCCCTCCCCGCCGCCCCCGAGGCGGCGAGTCGGGCGGGGCG
GATGATGGTCTCCCGTGGGCGGCTCGCCCCGCGGATGGCCGAAAGACGAG
CCTGCGGCGTGGATCGCCGCGACGGACGGTGGAAGAGCGAAACTT

Sample 6:

TGTGAATTGCAGAATCCCGtGAACcaTCGAGTCTTTGAACGCAAGTTGCGCC
CGAAGCCATTAGGCCGAGGGCACGTCTGCCTGGGTGTCACACAACGTCGC
CCCCTCCCCGCCGCCCCCGAGGCGGCGAGTCGGGCGGGGCGGATGATGGT
CTCCCGTGGGCGGCTCGCCCCGCGGATGGCCGAAAGACGAGCCTGCGGCG
TGGATCGCCGCGACGGACGGTGGAAGAGCGAAACCTCGAGACCGGTCGC
GCGCGAGTCCTCCCTTGGGGCAGGCTGCCAGACCCTTTAGCGTCGCCGCGT
ACGGCGGGCGCTTACGACGCGACCCCAGGTCAGGCGGGGCTACCCGCTGA
GTTTAAGCATATCAATAAGCGGAGGAAAAGAAACTAACCAGGATTCCCTTA
GTAACGGCGAGCGAACCGGgAAAAGCCCACCTtGAGAATCGGTCGCCCCCG
GCGTCCGAATTGTAGTCTG

Sample 7:

CGACGACTGGTTAGACCCTCGGGCGGGCGGAGGGCGCGCGTCGCCCCGAG
CCCCCCCTCCCGGGGACTCCCGCAGCCCCGCGCTGCCCGAGTCCGCCCGG
GAGAAATAACAAACCTCGGCGCCGGACGCGCCAAGGAACTCGAACGAAAT
AGCGCGCTCCCGGCGGCCCGGAGACGGTGCCCGCGCGGGGAGCGTCGCG
AGAATTGTATCCAAAACGACTCTCGGCAACGGATATCTCGGCTCTCGCATC
GATGAAGAACGTAGCGAAATGCGATACTTGGTGTGAATTGCAGAATCCCGT
GAACCATCGAGTCTTTGAACGCAAGTTGCGCCCGAAGCCATTAGGCCGAG
GGCACGTCTGCCTGGGTGTCACACAACGTCGCCCCCTCCCCGCCGCCCCC
GAGGCGGCGGGTCGGGCGGGGCGGATGATGGCCTCCCGTGGGCGAATCGC
CCCGCGGATGGCCGAAAGACGAGCCTGCGGCGTGGAACGCCGCGACGGA
CGGTGGAAGAGCGAACCTA

Sample 8:

AAATTTTTGCTCTTCCACCGTCCGTCGCGGCAGCCCACGCCGCAGGCTCGT
CTTTCGGCCATCCGCGGGGCGAGTCGCCCACGGGAGGCCATCGTCCGCCC
CGCCCGACGCGCCGCCTCGGGGGCGGCGGGGAGGGGGCGACGTTGCGTG

ACACCCAGGCAGACGTGCCCTCGGCCTAATGGCTTCGGGCGCAACTTGCG
TTCAAAGACTCGATGGTTCACGGGATTCTGCAATTCACACCAAGTATCGCA
TTTCGCTACGTTCTTCATCGATGCGAGAGCCGAGATATCCGTTGCCGAGAGT
CGTTTTGGATACAGTTCTCGCGACGCTCCCCGCGCGGGCACCGTCTCCGGG
CCGCCGAGAGCGCGCTCCTTCGTTCGAGTTCCTTGGCGCGTCCGGCGCCG
AGGTTTGTTATTCTGCCGGGCGGACTCGGGCAGCGCCAGGCTGCGGGAGT
CCCCGACAGGGGGGGCTCGGGGCGACGCGCGCCCTCCGCCCGCCCGAGG
GTCTAACCGGTTCGCGGGTCGTCCTTTTTGATAGGCATCGACAATGTAAC

Sample 9:

CATTGTCGATGCCTATCAAAAAGGACGACCCGCGAACCGGTTAGACCCTCG
GGCGGGCGGAGGGCGCGCGTCGCCCCGAGCCCCCCCTGTCGGGGACTCCC
GCAGCCTGGCGCTGCCCGAGTCCGCCCGGCAGAATAACAAACCTCGGCGC
CGGATGCGCCAAGGAACTCGAACGAAGGAGCGCGCTCTCGGCGGCCCGG
AGACGGTGCCCGCGCGGGGAGCGTCGCGAGAACTGTATCCAAAACGACTC
TCGGCAACGGATATCTCGGCTCTCGCATCGATGAAGAACGTAGCGAAATGC
GATACTTGGTGTGAATTGCAGAATCCCGTGAACCATCGAGTCTTTGAACGC
AAGTTGCGCCCGAAGCCATTAGGCCGAGGGCACGTCTGCCTGGGTGTCAC
GCAACGTCGCCCCCTCCCCGCCGCCCCCGAGGCGGCGCGTCGGGCGGGGC
GGACGATGGCCTCCCGTGGGCGACTCGCCCCGCGGATGGCCGAAAGACGA
GCCTGCGGCGTGGGCTGCCGCGACGGACGGTGGAAGAGCG

Sample 10:

CCTGGTGTGAATTGCAGAATCCCGTGAACCATCGAGTCTTTGAACGCAAGT
TGCGCCCGAAGCCATTAGGCCGAGGGCACGTCTGCCTGGGTGTCACACAA
CGTCGCCCCCTCCCCGCCGCCCCCGAGGCGGCGAGTCGGGCGGGGCGGAT
GATGGTCTCCCGTGGGCGGCTCGCCCCGCGGATGGCCGAAAGACGAGCCT
GCGGCGTGGATCGCCGCGACGGACGGTGGAAGAGCGAAACCTCGAGACC
GGTCGCGCGCGAGTCCTCCCTTGGGGCAGGCTGCCAGACCCTTTAGCGTC
GCCGCGTACGGCGGGCGCTTACGACGCGACCCCAGGTCAGGCGGGGCTAC
CCGCTGAGTTTAAGCATATCAATAAGCGGAGGAAAAGAAACTAACCAGGAT
TCCCTTAGTAACGGCGAGCGAACCGGGAAAAGCCCACCTTGAGAATCGGT
CGCCCCCGGCGTCCGAATTGTAGTCTGG

Sample 11:

AAATCTTGTGTGAATTGCAGAATCCCGTGAACCATCGAGTCTTTGAACGCA
AGTTGCGCCCGAAGCCATTAGGCCGAGGGCACGTCTGCCTGGGTGTCACG
CAACGTCGCCCCCTCCCCGCCGCCCCCGAGGCGGCGCGTCGGGCGGGGCG
GACGATGGCCTCCCGTGGGCGACTCGCCCCGCGGATGGCCGAAAGACGAG
CCTGCGGCGTGGGCTGCCGCGACGGACGGTGGAAGAGCGAAACCTCGAG
ACCGGTCGCGCGCGAGTCCTCCCTTGGGGCAGGCTGCGAGACCCTTTAGC
GTCGTCGCGTGTAGCGGGCGCTTACGATGCGACCCCAGGTCAGGCGGGGC
CACCCGCTGAGTTTAAGCATATCAATAAGCGGAGGAAAAGAAACTAACCA
GGATTCCCTTAGTAACGGCGAGCGAACCGGGAAAAGCCCACCTTGAGAAT
CGGTCGCCCCCGGCGTCCGAATTGTAGTCTGAAGAAAA

Sample 12:

TGGTGTGAATTGCAGAATCCCGTGAACCATCGAGTCTTTGAACGCAAGTTG
CGCCCGAAGCCATTAGGCCGAGGGCACGTCTGCCTGGGTGTCACGCAACG

TCGCCCCCTCCCCGCCGCCCCCGAGGCGGCGCGTCGGGCGGGGCGGACGA
TGGCCTCCCGTGGGCGACTCGCCCCGCGGATGGCCGAAAGACGAGCCTGC
GGCGTGGGCTGCCGCGACGGACGGTGGAAGAGCGAAACCTCGAGACCGG
TCGCGCGCGAGTCCTCCCTTGGGGCAGGCTGCGAGACCCTTTAGCGTCGTC
GCGTGTAGCGGGCGCTTACGATGCGACCCCAGGTCAGGCGGGGCCACCCG
CTGAGTTTAAGCATATCAATAAGCGGAGGAAAAGAAACTAACCAGGATTCC
CTTAGTAACGGCGAGCGAACCGGgAAAAGCCCACCTTGAGAATCGGTCGC
CCCCGGCGTCCGAATTGTAGTC

Sample 13:

TACAATTCGGACGCCGGGGGCGACCGATTCTCAAGGTGGGCTTTTCCCGGT
TCGCTTGCCGTTAATAAGGGAATCCTGGTTAGTTTCTTTTCCTCCGCTTATTG
ATATGCTTAAACTCAGCGGGTAGCCCCGCCTGACCTGGGGTCGCGTCGTAA
GCGCCCGCCGTACGCGGCGACGCTAAAGGGTCTGGCAGCCTGCCCCAAGG
GAGGACTCGCGCGCGACCGGTCTCGAGGTTTCGCTCTTCCACCGTCCGTCG
CGGCGATCCACGCCGCAGGCTCGTCTTTCGGCCATCCGCGGGGCGAGCCG
CCCACGGGAGACCATCATCCGCCCCGCCCGACTCGCCGCCTCGGGGGCGG
CGGGGAGGGGGCGACGTTGTGTGACACCCAGGCAGACGTGCCCTCGGCCT
AATGGCTTCGGGCGCAACTTGCGTTCAAAGACTCGATGGTTCACGGGATTC
TGCAATTCACACAAGTT

Sample 14:

GCGATATCTGGTGTGAATTGCAGAATCCCGTGAACCATCGAGTCTTTGAAC
GCAAGTTGCGCCCGAAGCCATTAGGCCGAGGGCACGTCTGCCTGGGCGTC
ACACATCGTTGCCCCCCCAACCACCGTGCCTCGCAAGAGGACGTGACGGG
ATGGGCGGATCATGGCCTCCCGCGGGCCTCGTCTCGCGGCTGGCCGAAATA
AGAGTCCTCGGTGGCGGTCGCCACGCTCCTCGGTGGACGAGTTTAACTCG
ATGCCGGTCGTGCGCGCGCCGTCACCTGGGCCGGGCTCGGAGACCCTGTC
GTGTCGCCCCGAGCGACAACGAAACCAACGCGACCCCAGGTCAGGCGGG
GCTACCCGCTGAGTTTAAGCATATCAATAAGCGGAGGAAAAGAAACTAACA
AGGATTCCCCTAGTAACGGCGAGCGAACCGGGAAAAGCCCACCATGAAAA
TCGGTCGCCCCAGGCGTCCCAATTGTAGT

Sample 15:

ATTGTCGATGCCTATCAAAAAGGACGACCCGCGaACCGGTTAGACCCTCGG
GCGGGCGGAGGGCGCGCGTCGCCCCGAGCCCCCCCTGTCGGGGACTCCCG
CAGCCTGGCGCTGCCCGAGTCCGCCCGGCAGAATAACAAACCTCGGCGCC
GGACGCGCCAAGGAACTCGAACGAAGGAGCGCGCTCTCGGCGGCCCGGA
GACGGTGCCCGCGCGGGGAGCGTCGCGAGAACTGTATCCAAAACGACTCT
CGGCAACGGATATCTCGGCTCTCGCATCGATGAAGAACGTAGCGAAATGCG
ATACTTGGTGTGAATTGCAGAATCCCGTGAACCATCGAGTCTTTGAACGCA
AGTTGCGCCCGAAGCCATTAGGCCGAGGGCACGTCTGCCTGGGTGTCACG
CAACGTCGCCCCCTCCCCGCCGCCCCCGAGGCGGCGCGTCGGGCGGGGCG
GACGATGGCCTCCCGTGGGCGACTCGCCCCGCGGATGGCCGAAAGACGAG
CCTGCGGCGTGGgcTGCCGCGACGGACGGTGGAAGAGCG

Sample 16:

AAGGTTTCGCTCTTCCACCGTCCGTCGCGGCAGCCCACGCCGCAGGCTCGT
CTTTCGGCCATCCGCGGGGCGAGTCGCCCACGGGAGGCCATCGTCCGCCC

CGCCCGACGCGCCGCCTCGGGGGCGGCGGGGAGGGGGCGACGTTGCGTG
ACACCCAGGCAGACGTGCCCTCGGCCTAATGGCTTCGGGCGCAACTTGCG
TTCAAAGACTCGATGGTTCACGGGATTCTGCAATTCACACCAAGTATCGCA
TTTCGCTACGTTCTTCATCGATGCGAGAGCCGAGATATCCGTTGCCGAGAGT
CGTTTTGGATACAGTTCTCGCGACGCTCCCCGCGCGGGCACCGTCTCCGGG
CCGCCGAGAGCGCGCTCCTTCGTTCGAGTTCCTTGGCGCGTCCGGCGCCG
AGGTTTGTTATTCTGCCGGGCGGACTCGGGCAGCGCCAGGCTGCGGGAGT
CCCCGACAGGGGGGGCTCGGGGCGACGCGCGCCCTCCGCCCGCCCGAGG
GTCTAACCGGTTCGCGGGTCGTCCTTTTTGATAGGCATCGACAATG

Sample 17:

GAATGATTCGCTCTTCCACCGTCCGTCGCGGCAGCCCACGCCGCAGGCTCG
TCTTTCGGCCATCCGCGGGGCGAGTCGCCCACGGGAGGCCATCGTCCGCCC
CGCCCGACGCGCCGCCTCGGGGGCGGCGGGGAGGGGGCGACGTTGCGTG
ACACCCAGGCAGACGTGCCCTCGGCCTAATGGCTTCGGGCGCAACTTGCG
TTCAAAGACTCGATGGTTCACGGGATTCTGCAATTCACACCAAGTATCGCA
TTTCGCTACGTTCTTCATCGATGCGAGAGCCGAGATATCCGTTGCCGAGAGT
CGTTTTGGATACAGTTCTCGCGACGCTCCCCGCGCGGGCACCGTCTCCGGG
CCGCCGAGAGCGCGCTCCTTCGTTCGAGTTCCTTGGCGCGTCCGGCGCCG
AGGTTTGTTATTCTGCCGGGCGGACTCGGGCAGCGCCAGGCTGCGGGAGT
CCCCGACAGGGGGGGCTCGGGGCGACGCGCGCCCTCCGCCCGCCCGAGG
GTCTAACCGGTTCGCGGGTCGTCCTTTTTGATAGGCATCGACAATGTTAC

Sample 18:

GATCATTGTCGATGCCTATCAAAAAGGAGGACCCGCGAACCGGTTAGACC
CTGGGGCGGGCGGAGGGCGTGCGTCGCCCCGAGCCCCCCCTCCCGGGGAC
TCCCGCAGCCGCGCGCTGCCCGAGTCCGCCCGGGAGAACAACAAACCTCG
GCGCCGGACGCGCCAAGGAACTCGAACGAAGGAGCGCGCTCCCGGCGGC
CCGGAGACGGCGCCCGCGCGGGGAGCGTCGCGAGAACTGTATCCAAAACG
ACTCTCGGCAACGGATATCTCGGCTCTCGCATCGATGAAGAACGTAGCGAA
ATGCGATACTTGGTGTGAATTGCAGAATCCCGTGAACCATCGAGTCTTTGA
ACGCAAGTTGCGCCCGAAGCCATTAGGCCGAGGGCACGTCTGCCTGGGTG
TCACACAACGTCGCCCCCTCCCCGCCGCCCCCGAGGCGGCGAGTCGGGCG
GGGCGGATGATGGTCTCCCGTGGGCGGCTCGCCCCGCGGATGGCCGAAAG
ACGAGCCTGCGGCGTGGATCGCCGCGACGGACGGTGGAAGAGCGAAACC
AT

Sample 19:

GAGGTTTCGCTCTTCCACCGTCCGTCGCGGCGTTCCACGCCGCAGGCTCGT
CTTTCGGCCATCCGCGGGGCGATTCGCCCACGGGAGGCCATCATCCGCCCC
GCCCGACCCGCCGCCTCGGGGGCGGCGGGGAGGGGGCGACGTTGTGTGA
CACCCAGGCAGACGTGCCCTCGGCCTAATGGCTTCGGGCGCAACTTGCGTT
CAAAGACTCGATGGTTCACGGGATTCTGCAATTCACACCAAGTATCGCATT
TCGCTACGTTCTTCATCGATGCGAGAGCCGAGATATCCGTTGCCGAGAGTC
GTTTTGGATACAATTCTCGCGACGCTCCCCGCGCGGGCACCGTCTCCGGGC
CGCCGGGAGCGCGCTATTTCGTTCGAGTTCCTTGGCGCGTCCGGCGCCGAG
GTTTGTTATTTCTCCCGGGCGGACTCGGGCAGCGCGGGGCTGCGGGAGTCC
CCGGGAGGGGGGGCTCGGGGCGACGCGCGCCCTCCGCCCGCCCGAGGGT
CTAACCGGTTCGCGGGTCGTCCTTTTTGATAGGCATCGACAATGATCC

Sample 20:

TCATTGTCGATGCCTATCAAAAAGGACGACCCGCGAACCGGTTAGACCCTC
GGGCGGGCGGAGGGCGCGCGTCGCCCCGAGCCCCCCCTCCCGGGGACTCC
CGCAGCCCCGCGCTGCCCGAGTCCGCCCGGGAGAAATAACAAACCTCGGC
GCCGGACGCGCCAAGGAACTCGAACGAAATAGCGCGCTCCCGGCGGCCCG
GAGACGGTGCCCGCGCGGGGAGCGTCGCGAGAATTGTATCCAAAACGACT
CTCGGCAACGGATATCTCGGCTCTCGCATCGATGAAGAACGTAGCGAAATG
CGATACTTGGTGTGAATTGCAGAATCCCGTGAACCATCGAGTCTTTGAACG
CAAGTTGCGCCCGAAGCCATTAGGCCGAGGGCACGTCTGCCTGGGTGTCA
CACAACGTCGCCCCCTCCCCGCCGCCCCCGAGGCGGCGGGTCGGGCGGGG
CGGATGATGGCCTCCCGTGGGCGAATCGCCCCGCGGATGGCCGAAAGACG
AGCCTGCGGCGTGGAACGCCGCGACGGACGGTGGAAGAGCGAAACTAC

Sample 21:

TTGTTATTGTCGATGCCTATCAAAAAGGAGGACCCGCGAACCGGTTAAGAC
CCTGGGGCGGGCGGAGGGCGTGCGTCGCCCCGAGCCCCCCCTCCCGGGGA
CTCCCGCAGCCGCGCGCTGTCCGAGTCCGCCCGGGAGAACAACAAACCTC
GGCGCCGGACGCGCCAAGGAACTCGAACGAAGGAGCGCGCTCCCGGCGG
CCCGGAGACGGCGCCCGCGCGGGGAGCGTCGCGAGAACTGTATCCAAAAC
GACTCTCGGCAACGGATATCTCGGCTCTCGCATCGATGAAGAACGTAGCGA
AATGCGATACTTGGTGTGAATTGCAGAATCCCGTGAACCATCGAGTCTTTG
AACGCAAGTTGCGCCCGAAGCCATTAGGCCGAGGGCACGTCTGCCTGGGT
GTCACACAACGTCGCCCCCTCCCCGCCGCCCCCGAGGCGGCGAGTCGGGC
GGGGCGGATGATGGTCTCCCGTGGGCGGCTCGCCCCGCGGATGGCCGAAA
GACGAGCCTGCGGCGTGGATCGCCGCGACGGACGGTGGAAGAGCGAAAA
CAT

Sample 22:

AGGTTCGCTCTTCCACCGTCCGTCGCGGCAGCCCACGCCGCAGGCTCGTCT
TTCGGCCATCCGCGGGGCGAGTCGCCCACGGGAGGCCATCGTCCGCCCCG
CCCGACGCGCCGCCTCGGGGGCGGCGGGGAGGGGGCGACGTTGCGTGAC
ACCCAGGCAGACGTGCCCTCGGCCTAATGGCTTCGGGCGCAACTTGCGTTC
AAAGACTCGATGGTTCACGGGATTCTGCAATTCACACCAAGTATCGCATTT
CGCTACGTTCTTCATCGATGCGAGAGCCGAGATATCCGTTGCCGAGAGTCG
TTTTGGATACAGTTCTCGCGACGCTCCCCGCGCGGGCACCGTCTCCGGGCC
GCCGAGAGCGCGCTCCTTCGTTCGAGTTCCTTGGCGCGTCCGGCGCCGAG
GTTTGTTATTCTGCCGGGCGGACTCGGGCAGCGCCAGGCTGCGGGAGTCCC
CGACAGGGGGGGCTCGGGGCGACGCGCGCCCTCCGCCCGCCCGAGGGTCT
AACCGGTTCGCGGGTCGTCCTTTTTGATAGGCATCGACAATAATC

Sample 23:

AAGGTTTCGCTCTTCCACCGTCCGTCGCGGCGATCCACGCCGCAGGCTCGT
CTTTCGGCCATCCGCGGGGCGAGCCGCCCACGGGAGACCATCATCCGCCCC
GCCCGACTCGCCGCCTCGGGGGCGGCGGGGAGGGGGCGACGTTGTGTGA
CACCCAGGCAGACGTGCCCTCGGCCTAATGGCTTCGGGCGCAACTTGCGTT
CAAAGACTCGATGGTTCACGGGATTCTGCAATTCACACCAAGTATCGCATT
TCGCTACGTTCTTCATCGATGCGAGAGCCGAGATATCCGTTGCCGAGAGTC
GTTTTGGATACAGTTCTCGCGACGCTCCCCGCGCGGGCGCCGTCTCCGGGC

CGCCGGGAGCGCGCTCCTTCGTTCGAGTTCCTTGGCGCGTCCGGCGCCGA
GGTTTGTTGTTCTCCCGGGCGGACTCGGACAGCGCGGCTGCGGGAGTC
CCCGGGAGGGGGGGCTCGGGGCGACGCACGCCCTCCGCCCGCCCCAGGG
TCTTAACCGGTTCGCGGGTCCTCCTTTTTGATAGGCATCGACAATAA

Sample 24:

TCGGGCGGGCGGAGGGCGCGCGTCGCCCCGAGCCCCCCCTGCCGGGGACT
CCCGCAGCCCCGCGCTGCCCGAGTCCGCCCGGGAGAAATAACAAACCTCG
GCGCCGGACGCGCCAAGGAACTCGAACGAAATAGCGCGCTCCCGGCGGCC
CGGAGACGGTGCCCGCGCGGGGAGCGTCGCGAGAATTGTATCCAAAACGA
CTCTCGGCAACGGATATCTCGGCTCTCGCATCGATGAAGAACGTAGCGAAA
TGCGATACTTGGTGTGAATTGCAGAATCCCGTGAACCATCGAGTCTTTGAA
CGCAAGTTGCGCCCGAAGCCATTAGGCCGAGGGCACGTCTGCCTGGGTGT
CACACAACGTCGCCCCCTCCCCGCCGCCCCCGAGGCGGCGGGTCGGGCGG
GGCGGATGATGGCCTCCCGTGGGCGACTCGCCCCGCGGATGGCCGAAAGA
CGAGC

Sample 25:

TCGATGCCTATCAAAAAGGACGACCCGCGAACCGGTTAGACCCTCGGGCG
GGCGGAGGGCGCGCGTCGCCCCGAGCCCCCCCTCCCGGGGACTCCCGCAG
CCCCGCGCTGCCCGAGTCCGCCCGGGAGAAATAACAAACCTCGGCGCCGG
ACGCGCCAAGGAACTCGAACGAAATAGCGCGCTCCCGGCGGCCCGGAGAC
GGTGCCCGCGCGGGGAGCGTCGCGAGAATTGTATCCAAAACGACTCTCGG
CAACGGATATCTCGGCTCTCGCATCGATGAAGAACGTAGCGAAATGCGATA
CTTGGTGTGAATTGCAGAATCCCGTGAACCATCGAGTCTTTGAACGCAAGT
TGCGCCCGAAGCCATTAGGCCGAGGGCACGTCTGCCTGGGTGTCACACAA
CGTCGCCCCCTCCCCGCCGCCCCCGAGGCGGCGGGTCGGGCGGGGCGGAT
GATGGCCTCCCGTGGGCGAATCGCCCCGCGGATGGCCGAAAGACGAGCCT
GCGGCGTGGAACGCCGCGACGGACGGTGGAAGAGC

Sample 26:

No sequences were obtained.

Sample 27:

ATGTTTCGCTCTTCCACCGTCCGTCGCGGCAGCCCACGCCGCAGGCTCGTC
TTTCGGCCATCCGCGGGGCGAGTCGCCCACGGGAGGCCATCGTCCGCCCC
GCCCGACGCGCCGCCTCGGGGGCGGCGGGGAGGGGGCGACGTTGCGTGA
CACCCAGGCAGACGTGCCCTCGGCCTAATGGCTTCGGGCGCAACTTGCGTT
CAAAGACTCGATGGTTCACGGGATTCTGCAATTCACACCAAGTATCGCATT
TCGCTACGTTCTTCATCGATGCGAGAGCCGAGATATCCGTTGCCGAGAGTC
GTTTTGGATACAGTTCTCGCGACGCTCCCCGCGCGGGCACCGTCTCCGGGC
CGCCGAGAGCGCGCTCCTTCGTTCGAGTTCCTTGGCGCGTCCGGCGCCGA
GGTTTGTTATTCTGCCGGGCGGACTCGGGCAGCGCCAGGCTGCGGGAGTC
CCCGACAGGGGGGGGCTCGGGGCGACGCGCGCCCTCCGCCCGCCCGAGGG
TCTAACCGGTTCGCGGGTCGTCCTTTTTGATAGGCATCGACAATGATC

Sample 28:

TTGTCGATGCCTATCAAAAAGGACGACCCGCGAACCGGTTAGACCCTCGG
GCGGGCGGAGGGCGCGCGTCGCCCCGAGCCCCCCCTCCCGGGGACTCCCG

CAGCCCCGCGCTGCCCGAGTCCGCCCGGGAGAAATAACAAACCTCGGCGC
CGGACGCGCCAAGGAACTCGAACGAAATAGCGCGCTCCCGGCGGCCCGGA
GACGGTGCCCGCGCGGGGAGCGTCGCGAGAATTGTATCCAAAACGACTCT
CGGCAACGGATATCTCGGCTCTCGCATCGATGAAGAACGTAGCGAAATGCG
ATACTTGGTGTGAATTGCAGAATCCCGTGAACCATCGAGTCTTTGAACGCA
AGTTGCGCCCGAAGCCATTAGGCCGAGGGCACGTCTGCCTGGGTGTCACA
CAACGTCGCCCCCTCCCCGCCGCCCCCGAGGCGGCGGGTCGGGCGGGGCG
GATGATGGCCTCCCGTGGGCGAATCGCCCCGCGGATGGCCGAAAGACGAG
CCTGCGGCGTGGAACGCCGCGACGGACGGTGGAAGAGCGAA

Sample 29:

No sequences were obtained.

Sample 30:

ACATTGTCGATGCCTATCAAAAAGGAGGACCTGCGAACCGGTTAAGACCCT
GGGGCGGGCGGAGGGCGTGCGTCGCCCCGAGCCCCCCCTCCCGGGGACTC
CCGCAGCCGCGCGCTGTCCGAGTCCGCCCGGGAGAACAACAAACCTCGGC
GCCGGACGCGCCAAGGAACTCGAACGAAGGAGCGCGCTCCCGGCGGCCC
GGAGACGGCGCCCGCGCGGGGAGCGTCGCGAGAACTGTATCCAAAACGA
CTCTCGGCAACGGATATCTCGGCTCTCGCATCGATGAAGAACGTAGCGAAA
TGCGATACTTGGTGTGAATTGCAGAATCCCGTGAACCATCGAGTCTTTGAA
CGCAAGTTGCGCCCGAAGCCATTAGGCCGAGGGCACGTCTGCCTGGGTGT
CACACAACGTCGCCCCCTCCCCGCCGCCCCCGAGGCGGCGAGTCGGGCGG
GGCGGATGATGGTCTCCCGTGGGCGGCTCGCCCCGCGGATGGCCGAAAGA
CGAGCCTGCGGCGTGGATCGCCGCGACGGACGGTGGAAGAGCG
